# Supplementary material for: Vitamin D and the Risks of Depression and Anxiety: An Observational Analysis and Genome-Wide Environment Interaction Study
Source: Nutrients. 2021 Sep 24;13(10):3343. doi: 10.3390/nu13103343 (PMC8538638; doi:10.3390/nu13103343)
Supplement: Supplementary file 1 [file nutrients-13-03343-s001.zip › nutrients-1374859-supplementary.pdf]

The Supplementary Information

Appendix: Questionnaire wording and format

Introduction

Table S1: Present and past depression and/or bipolar affective disorder.

Table S2: Generalized anxiety disorder.

Table S1: present and past depression and/or bipolar affective disorder.		
Patient health questionnaire (PHQ) scores:		
a) 20514<br>b) 20510<br>c) 20534<br>d) 20519<br>e) 20511<br>f) 20507<br>g) 20508<br>h) 20518<br>i) 20513	Over the last 2 weeks, how often have you been bothered by any of the following problems?<br>a. Little interest or pleasure in doing things<br>b. Feeling down, depressed, or hopeless<br>c. Trouble falling or staying asleep, or sleeping too much<br>d. Feeling tired or having little energy<br>e. Poor appetite or overeating<br>f. Feeling bad about yourself or that you are a failure or have let yourself or your family down<br>g. Trouble concentrating on things, such as reading the newspaper or watching television<br>h. Moving or speaking so slowly that other people could have noticed? Or the opposite — being so fidgety or restless that you have been moving around a lot more than usual<br>i. Thoughts that you would be better off dead or of hurting yourself in some way	[Select one from the following for each of the statements]<br>- 01 Not at all<br>- 02 Several days<br>- 03 More than half the days<br>- 04 Nearly every day<br>- DA Prefer not to answer
Depressive symptoms:		
20446	Have you ever had a time in your life when you felt sad, blue, or depressed for two weeks or more in a row?	[Select one from]<br>- 01 Yes<br>- 00 No<br>- DA Prefer not to answer
20441	Have you ever had a time in your life lasting two weeks or more when you lost interest in most things like hobbies, work, or activities that usually give you pleasure?	[Select one from]<br>- 01 Yes<br>- 00 No<br>- DA Prefer not to answer
Depression single episode:		

20447	Did this worst period start within two months of the death of someone close to you or after a stressful or traumatic event in your life?	[Select one from]<br>- 01 Yes<br>- 00 No<br>-DA Prefer not to answer
20436(Fraction of day affected)	How much of the day did these feelings usually last?	- 04 All day long<br>- 03 Most of the day<br>- 02 About half of the day<br>- 01 Less than half of the day<br>- NA Do not know<br>- DA Prefer not to answer
20439 (Frequency of depressed days)	Did you feel this way	- 03 Every day<br>- 02 Almost every day<br>- 01 Less often<br>- NA Do not know<br>- DA Prefer not to answer
20449	Did you feel more tired out or low on energy than is usual for you?	- 01 Yes<br>- 00 No<br>- NA Do not know<br>- DA Prefer not to answer
20536	Did you gain or lose weight without trying, or did you stay about the same weight?	- 01 Gained weight<br>- 02 Lost weight<br>- 03 Both gained and lost some weight during the episode<br>- 00 Stayed about the same or was on a diet<br>- NA Do not know<br>- DA Prefer not to answer
20532	Did your sleep change?	- 01 Yes<br>- 00 No<br>- NA Do not know<br>- DA Prefer not to answer
a) 20533<br>b) 20535	Was that:<br>a Trouble falling asleep	[Three questions grouped together,

c) 20534	b Waking too early<br>c Sleeping too much	each with forced choice]<br>- 01 Yes<br>- 00 No
20435	Did you have a lot more trouble concentrating than usual?	- 01 Yes<br>- 00 No<br>- NA Do not know<br>- DA Prefer not to answer
20450	People sometimes feel down on themselves, no good, worthless. Did you feel this way?	- 01 Yes<br>- 00 No<br>- NA Do not know<br>- DA Prefer not to answer
20437	Did you think a lot about death – either your own, someone else's or death in general?	- 01 Yes<br>- 00 No<br>- UN Do not know<br>- DA Prefer not to answer
20438 (Duration of worst depression)	About how long altogether did you feel this way? Count the time before, during and after the worst two weeks.	- 01 Less than a month<br>- 02 Between one and three months<br>- 03 Over three months, but less than six months<br>- 04 Over six months, but less than 12 months<br>- 05 One to two years<br>- 06 Over two years<br>- DA Prefer not to answer
20440	Think about your roles at the time of this episode, including study / employment, childcare and housework, leisure pursuits. How much did these problems interfere with your life or activities?	- 03 A lot<br>- 02 Somewhat<br>- 01 A little<br>- 00 Not at all<br>- DA Prefer not to answer
	Regarding times in your life when you have had feelings of depression or loss of interest:	Display throughout following questions B15 to B20
20442	How many periods did you have in your life lasting two or more weeks where you felt like	- 01 One<br>- 02 Several

	this?	- DA Prefer not to answer
20442	Enter number	BBOX1: Integer box 2 – 999 BBOX1 & "number of times"<br>OR<br>- 01 Too many to count / One episode ran into the next.
20433	About how old were you the FIRST time you had a period of two weeks like this? (Whether or not you received any help for it.)	BBOX2: Integer box 2 to current age BBOX2 & "years of age when first felt this way"<br>OR<br>- UN Do not know<br>OR<br>- DA Prefer not to answer
20445	Did this episode occur within months of giving birth? Or has it been suggested you had post-natal depression?	- 01 Yes<br>- 00 No<br>- NA Not applicable<br>- UN Do not know<br>- DA Prefer not to answer
20434	About how old were you the LAST time you had a period of two weeks like this? (Whether or not you received any help for it)	BBOX3: Integer box 2 to current age BBOX3 & "years of age when last felt this way"<br>OR<br>- UN Don't know<br>OR<br>- DA Prefer not to answer
20448	Did you ever tell a professional about these problems (medical doctor, psychologist, social worker, counsellor, nurse, clergy, or other helping professional)?	- 01 Yes<br>- 00 No<br>- UN Do not know<br>- DA Prefer not to answer

20546	Did you ever try the following for these problems? (tick all that apply)	[Select up to three]<br>- 01 Medication prescribed to you (for at least two weeks)<br>- 02 Unprescribed medication (more than once)<br>- 03 Drugs or alcohol (more than once)<br>- 00 None of the above<br>- DA Prefer not to say
20547	Did you ever try talking therapies for these problems, or other structured activities you regard as therapeutic? Include only those you attended more than once.	[Select up to two]<br>- 01 Talking therapies, such as psychotherapy, counselling, group therapy or CBT<br>- 02 Other therapeutic activities such as mindfulness, yoga or art classes<br>- 00 None of the above<br>- DA Prefer not to answer
	Now we want to know about some different symptoms.	- Next

Table S2: Generalized anxiety disorder (GAD).		
GAD-7		
a) 20506<br>b) 20509<br>c) 20520<br>d) 20515<br>e) 20516<br>f) 20505<br>g) 20512	Over the last 2 weeks, how often have you been bothered by any of the following problems?<br><br>a) Feeling nervous, anxious or on edge<br>b) Not being able to stop or control worrying<br>c) Worrying too much about different things<br>d) Trouble relaxing<br>e) Being so restless that it is hard to sit still<br>f) Becoming easily annoyed or irritable<br>g) Feeling afraid as if something awful might happen<br><br>[7 questions on one screen in grid]	[Select one from the following for each of the statements]<br>- 01 Not at all - 02 Several days - 03 More than half the days - 04 Nearly every day - DA Prefer not to answer
GAD ever		
20421	Have you ever had a period lasting one month or longer when most of the time you felt worried, tense, or anxious?	- 01 Yes<br>- 00 No<br>- UN Do not know<br>- DA Prefer not to answer
20420	What is the longest period of time that this kind of worrying has ever continued?	Cbox2: Integer 0-99<br>Cbox1: Integer 0-11<br>Cbox02 & "year(s) and" & Cbox01 &"month(s)"<br>OR<br>- 03 All my life / as long as I can remember
20425	People differ a lot in how much they worry about things. Did you ever have a time when you worried a lot more than most people would in your situation?	- 01 Yes<br>- 00 No<br>- UN Do not know<br>- DA Prefer not to answer
	Please think of the period in your life when you have felt worried, tense, anxious, or more worried than most people would in your situation. This could be in the past, or it could be continuing now.	Display throughout following questions C4 to C10

20542	During that period, was your worry stronger than in other people?	- 01 Yes<br>- 00 No<br>- UN Do not know<br>- DA Prefer not to answer
20538	Did you worry most days?	- 01 Yes<br>- 00 No<br>- UN Do not know<br>- DA Prefer not to answer
20543	Did you usually worry about one particular thing, such as your job security or the failing health of a loved one, or more than one thing?	- 01 One thing<br>- 02 More than one thing<br>- UN Do not know<br>- DA Prefer not to answer
20541	Did you find it difficult to stop worrying?	- 01 Yes<br>- 00 No<br>- UN Do not know<br>- DA Prefer not to answer
20540	Did you ever have different worries on your mind at the same time?	- 01 Yes<br>- 00 No<br>- UN Do not know<br>- DA Prefer not to answer
20539	How often was your worry so strong that you couldn't put it out of your mind no matter how hard you tried?	- 03 Often<br>- 02 Sometimes<br>- 01 Rarely<br>- 00 Never<br>- UN Do not know<br>- DA Prefer not to answer
20537	How often did you find it difficult to control your worry?	- 03 Often<br>- 02 Sometimes<br>- 01 Rarely<br>- 00 Never<br>- UN Do not know<br>- DA Prefer not to answer

a) 20426<br>b) 20423<br>c) 20429<br>d) 20419<br>e) 20422<br>f) 20417<br>g) 20427	When you were worried or anxious, were you also:<br><br>a) Restless?<br>b) Keyed up or on edge?<br>c) Easily tired?<br>d) Having difficulty keeping your mind on what you were doing?<br>e) More irritable than usual?<br>f) Having tense, sore, or aching muscles?<br>g) Often having trouble falling or staying asleep?<br><br>[Seven questions on one screen]	Force choice:<br>- 01 Yes<br>- 02 No<br>- NA Do not know<br>For following options:
20428	Did you ever tell a professional about these problems (medical doctor, psychologist, social worker, counsellor, nurse, clergy, or other helping professional)?	- 01 Yes<br>- 00 No<br>- UN Do not know<br>- DA Prefer not to answer
20549	Did you ever use the following for the worry or the problems it caused? (tick all that apply):<br><br>Please include any treatments that you have already told us about under 'depression' if they were also for anxiety:	- 01 Medication prescribed to you (for at least two weeks)<br>- 02 Unprescribed medication (more than once)<br>- 03 Drugs or alcohol (more than once)<br>- 00 None of the above<br>- DA Prefer not to say
20550	Did you ever try talking therapies for these problems, or other structured activities you regard as therapeutic? Include only those you attended more than once. Please include any treatments that you have already told us about under "depression" if they were also for anxiety:	[Select up to two]<br>- 01 Talking therapies, such as psychotherapy, counselling, group therapy or CBT<br>- 02 Other therapeutic activities such as mindfulness, yoga or art classes<br>- 00 None of the

		above<br>- DA Prefer not to answer
20418	Think about your roles at the time of this episode, including study / employment, childcare and housework, leisure pursuits. How much did these problems interfere with your life or activities?	[Choose one of]<br>- 03 A lot<br>- 02 Somewhat<br>- 01 A little<br>- 00 Not at all<br>- DA Prefer not to answer
